# Epigenetic and Neuronal Activity Markers Suggest the Recruitment of the Prefrontal Cortex and Hippocampus in the Three-Hit Model of Depression in Male PACAP Heterozygous Mice

**DOI:** 10.3390/ijms231911739

**Published:** 2022-10-03

**Authors:** Tamás Gaszner, József Farkas, Dániel Kun, Balázs Ujvári, Nóra Füredi, László Ákos Kovács, Hitoshi Hashimoto, Dóra Reglődi, Viktória Kormos, Balázs Gaszner

**Affiliations:** 1 Department of Anatomy, Medical School, University of Pécs, H-7624 Pécs, Hungary; 2 Research Group for Mood Disorders, Centre for Neuroscience Medical School, University of Pécs, H-7624 Pécs, Hungary; 3 Laboratory of Molecular Neuropharmacology, Graduate School of Pharmaceutical Sciences, Osaka University, 1-6 Yamadaoka, Suita 565-0871, Osaka, Japan; 4 Molecular Research Center for Children’s Mental Development, United Graduate School of Child Development, Osaka University, Kanazawa University, Hamamatsu University School of Medicine, Chiba University and University of Fukui, 2-2 Yamadaoka, Suita 565-0871, Osaka, Japan; 5 Division of Bioscience, Institute for Datability Science, Osaka University, 1-1 Yamadaoka, Suita 565-0871, Osaka, Japan; 6 Transdimensional Life Imaging Division, Institute for Open and Transdisciplinary Research Initiatives, Osaka University, 2-1 Yamadaoka, Suita 565-0871, Osaka, Japan; 7 Department of Molecular Pharmaceutical Sciences, Graduate School of Medicine, Osaka University, 2-2 Yamadaoka, Suita 565-0871, Osaka, Japan; 8 ELKH-PTE PACAP Research Group Department of Anatomy, Medical School, University of Pécs, H-7624 Pécs, Hungary; 9 Department of Pharmacology and Pharmacotherapy, University of Pécs, H-7624 Pécs, Hungary

**Keywords:** histone acetylation, H3K9ac, FOSB, chronic stress, maternal deprivation

## Abstract

Depression and its increasing prevalence challenge patients, the healthcare system, and the economy. We recently created a mouse model based on the three-hit concept of depression. As genetic predisposition (first hit), we applied pituitary adenylate cyclase-activating polypeptide heterozygous mice on CD1 background. Maternal deprivation modeled the epigenetic factor (second hit), and the chronic variable mild stress was the environmental factor (third hit). Fluoxetine treatment was applied to test the predictive validity of our model. We aimed to examine the dynamics of the epigenetic marker acetyl-lysine 9 H3 histone (H3K9ac) and the neuronal activity marker FOSB in the prefrontal cortex (PFC) and hippocampus. Fluoxetine decreased H3K9ac in PFC in non-deprived animals, but a history of maternal deprivation abolished the effect of stress and SSRI treatment on H3K9ac immunoreactivity. In the hippocampus, stress decreased, while SSRI increased H3K9ac immunosignal, unlike in the deprived mice, where the opposite effect was detected. FOSB in stress was stimulated by fluoxetine in the PFC, while it was inhibited in the hippocampus. The FOSB immunoreactivity was almost completely abolished in the hippocampus of the deprived mice. This study showed that FOSB and H3K9ac were modulated in a territory-specific manner by early life adversities and later life stress interacting with the effect of fluoxetine therapy supporting the reliability of our model.

## 1. Introduction

Major depression, with its strikingly increasing prevalence, challenges the affected people, their families, the healthcare system, and even the economy [1]. Basic neuroscience has to helped to answer the complex question of how to find new prevention and/or therapeutic strategies for mood disorders. In order to deepen our understanding of a complex disease, with a highly complex multifactorial background [2], a complex animal model is needed in basic research. To create a reliable animal model, we decided to follow the three-hit concept of the disease. According to this, the co-incidence of (a) genetic predisposition, (b) epigenetic factors, and (c) stress exposure may precipitate the symptoms [3,4]. We applied mice carrying a genetic alteration by lacking one functional allele of pituitary adenylate cyclase-activating polypeptide (PACAP) on CD1 background [5,6]. We decided to use these heterozygous (HZ) mice because they exhibited reduced PACAP levels in the brain [7] associated with a depression-like phenotype [8], and the role of PACAP has been shown in multiple aspects of stress adaptation response, including the activity of higher-order limbic centers [8,9] and the regulation of the hypothalamus–pituitary–adrenal (HPA) axis [6,8,9,10,11,12,13,14,15,16,17,18,19,20]. 

In our model, PACAP HZ mice were exposed to maternal deprivation (second hit) that evokes epigenetic changes [6,21] in the vulnerable early life period [22,23], thus increasing the risk to develop a depression-like state in animal models [24,25], in line with human observations [26]. Chronic variable mild stress (CVMS) is also commonly used to induce a depression-like state in rodent models [27]. Therefore, we incorporated this paradigm as the third “hit” and successfully verified the classical Willnerian [28] construct and the face validity of our model [5,6]. Most recently, we also tested the predictive validity of our model by applying fluoxetine, a standard selective serotonin reuptake inhibitor (SSRI) treatment, in our mice carrying all three risk factors in the forced swim, tail suspension, marble burying, and light–dark box tests [21]. We observed that the treatment reversed the increased anxiety and depression-like behavior in PACAP HZ mice that carry all three risk factors [21]. We also recognized that the therapeutic effect on functional–morphological alterations in the bed nucleus of the stria terminalis and central amygdala, and, in brainstem centers for mood control (i.e., the ventral tegmental area (VTA), centrally projecting the Edinger–Westphal nucleus and the dorsal raphe nucleus) depended on the existence of the epigenetic hit, maternal deprivation [21]. In our most recent work [21], we did not examine all the important players of mood control and stress adaptation response. Considering the fact that major depression has a deep impact on higher-order limbic centers [29,30], we decided to test our model in further limbic areas. 

The prefrontal cortex (PFC) is one of the most important brain territories in higher-order cognitive functions and behavior control [31]. The question of how this brain area contributes to the pathogenesis of depression has been investigated for a long time [32,33], including through functional imaging [34], studies on individuals who suffered damage of the PFC [35], and reports on cases treated by deep brain stimulation [36,37]. Functional neuroanatomical studies in rodent models consonantly suggest the complex contribution of the PFC to the psychopathology of depression recruiting serotonergic [38], GABAergic [39], and glutamatergic [40] mechanisms. Molecular studies proved the recruitment of brain-derived neurotrophic-factor-induced intracellular signaling via cAMP response element binding protein [41,42] and the contribution of epigenetic mechanisms [43] in models of depression. Studies on early life adversity have also shown that maternal deprivation altered cognitive functions and exaggerated plasticity [44]. Chronic environmental stress in the PFC of adult animals caused an elevation of microglia activity and induced an increased neuronal activity, as assessed by FOSB immunoreactivity (ir) [45]. FOSB is a protein product of the Finkel–Biskis–Jinkins murine sarcoma virus-related cellular oncogene, and it is a commonly used functional–morphological indicator of chronic neuronal activation [46,47]. Further, efferent PFC projections also suggest that the altered PFC activity may contribute to the behavioral changes attributed to other limbic centers, including the amygdala, VTA, and hippocampus [48].

The hippocampal formation is probably the most often examined brain territory in depression [29,49]. It is known that stress may affect hippocampal neuronal plasticity, which also contributes to the development of depression [41,42,43,50,51]. Stress exposure reduces the hippocampal volume and activity, as found in the majority of patients who suffer from depression; however, it is not clear whether these phenomena are the consequences of depression, or they should be considered predisposing factors [52,53]. Hippocampal connections with the HPA axis, PFC, and extended amygdala also suggest its central role in stress adaptation response and mood control [54,55]. The three main parts of the hippocampus, the areas of cornu ammonis (CA) 1, CA3, and dentate gyrus (DG), show decreased levels of brain-derived neurotrophic factor upon maternal separation, which was reversible by antidepressant treatment [56]. The elevation of FOSB-ir upon chronic stress was also reversible by antidepressant treatment in our earlier work performed in PACAP knockout (KO) mice [9]. Hippocampal GABAergic interneurons are also sensitive to early life stress affecting the epigenome, as they exert increased DNA methylation upon stress, associated with depression-like behavior in mice [57].

Based on these, in order to further support the validity of our model and to gain deeper insight into the functional–neuromorphological changes that underlie depression, in this study, we decided to focus on the recruitment of the PFC and the CA1 and CA3, as well as DG of the hippocampus. The altered histone acetylation pattern is a commonly used indicator of long-lasting regulatory changes in epigenetic alterations in depression models [58,59,60]. FOSB is also a reliable marker for long-term changes in neuronal activity in depression models [47,61]. Therefore, we aimed to test if the (a) epigenetic marker acetyl-lysine 9 H3 histone (H3K9ac) and (b) the chronic neuronal activity marker FOSB are affected in these brain regions in our model, and how these changes are influenced by fluoxetine therapy. We hypothesized that, as risk factors, the hits would interact with each other and with the fluoxetine treatment, as mirrored by the altered H3 histone acetylation and the FOSB neuronal activity pattern.

## 2. Results

### 2.1. H3K9ac Immunoreactivity

In both areas of interest in all of our experimental groups, we successfully detected a strong nuclear H3K9ac-ir. We compared the number of immunoreactive cells in order to assess the effect of the quality of maternal care, stress exposure, and SSRI treatment on this epigenetic marker.

#### 2.1.1. Prefrontal Cortex

**Figure 1:**
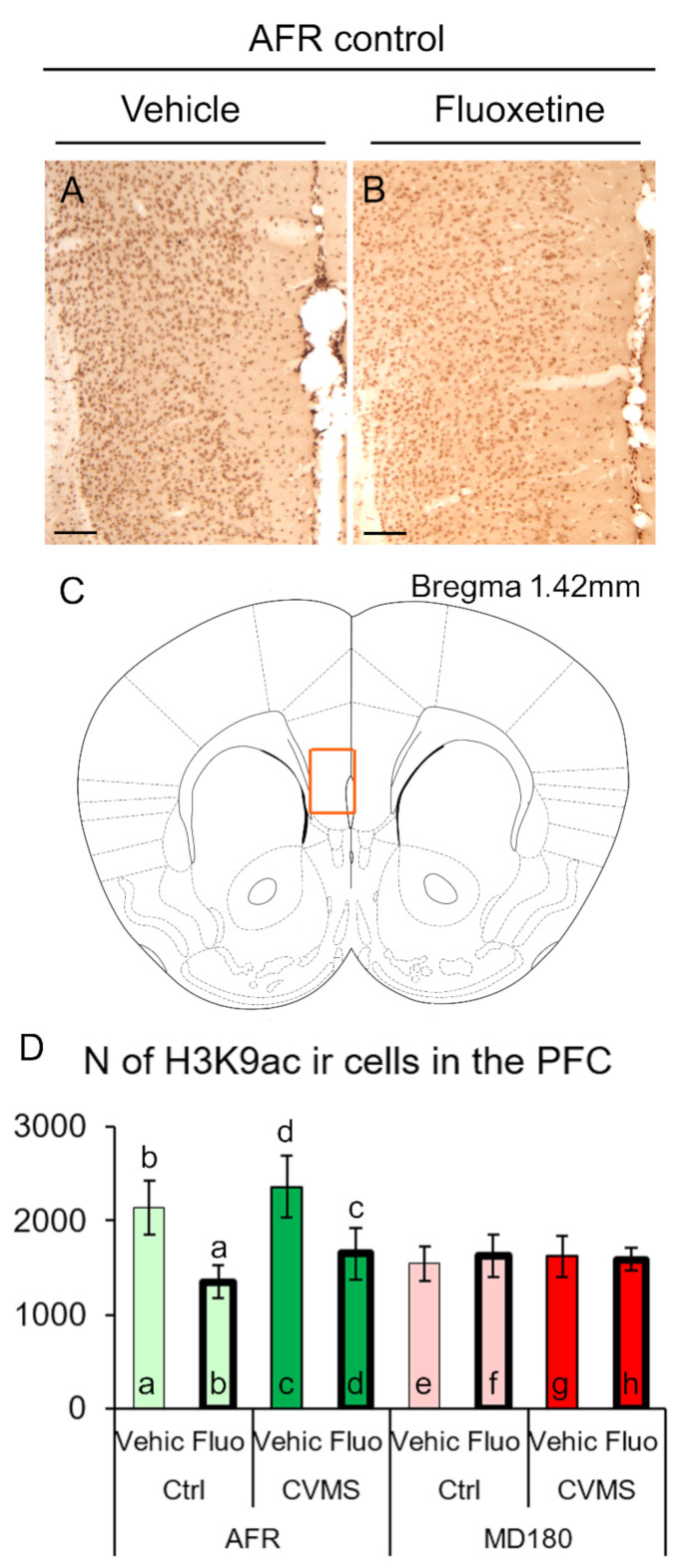
Acetyl-lysine 9 H3 histone (H3K9ac) immunoreactivity (ir) in the prefrontal cortex (PFC). Representative images illustrating H3K9ac-ir nuclei in the PFC of vehicle (Vehic) (**A**) and fluoxetine (Fluo)-treated PACAP heterozygous control (Ctrl) AFR mice. The imaged area shown in (**A**,**B**) corresponds to highlighted PFC region in (**C**) (scheme modified after Paxinos and Franklin [62]). Histogram (**D**) illustrates the dynamics of H3K9ac-ir cell counts. Lettering indicates the most relevant significant differences between pairs of groups, according to the post hoc tests (n = 4–6). AFR: animal facility rearing, CVMS: chronic variable mild stress, MD180: 180 min maternal deprivation. Bars: 100 µm.

MANOVA revealed that the main effect of the fluoxetine treatment (F_1,32_ = 7.72; *p* = 0.039) and the interaction of maternal care and treatment (F_1,32_ = 5.43; *p* = 0.027) influenced the H3K9ac immunosignal significantly. Post hoc tests showed that the fluoxetine treatment reduced the number of H3K9ac immunopositive cells in the animal facility-reared (AFR) control group (compare Figure 1A and Figure 1B, as well as bars “a” and “b” in Figure 1D, *p* = 0.036). In line with this, in CVMS-exposed mice, fluoxetine treatment was associated with a reduced number of H3K9ac-ir cells (bars “c” and “d” in Figure 1D, *p* = 0.047). Importantly, in the control vehicle-treated mice with MD180 history, a tendentiously lower cell count was detected (compare bars “a” and “e” in Figure 1D, *p* = 0.068), and the fluoxetine treatment did not affect the H3K9ac-ir, in the mice that experienced maternal deprivation (bars “e–h” in Figure 1D).

#### 2.1.2. Hippocampus

In order to test the possible subdivision-specific epigenetic effects in our model on the hippocampus, we assessed the CA1 and CA3 regions, and DG.

**Figure 2:**
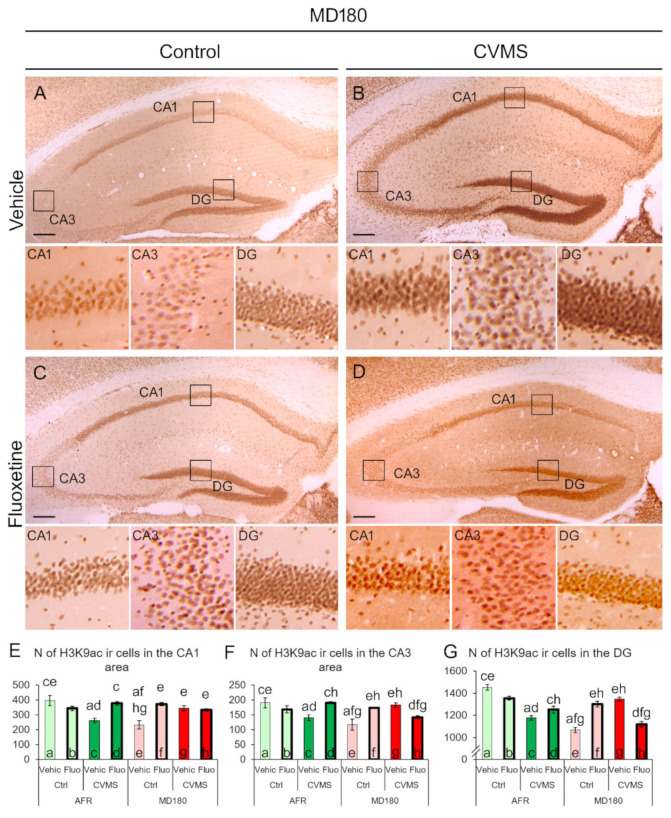
Acetyl-lysine 9 H3 histone (H3K9ac) immunoreactivity (ir) in the cornu ammonis (CA) 1, CA3, and dentate gyrus (DG) of the hippocampus. Representative images illustrate the H3K9ac-ir in vehicle (Vehic) (**A**) and fluoxetine (Fluo)-injected (**C**) control (Ctrl) and chronic variable mild stress (CVMS)-exposed vehicle (**B**) and fluoxetine-injected (**D**) mice with a history of maternal deprivation (MD180). Boxed areas are shown in higher magnification insets below the respective low magnification image. Histograms illustrate the dynamics of H3K9ac-ir in the CA1 (**E**), CA3 (**F**), and DG (**G**). Lettering indicates the most relevant significant differences between pairs of groups, according to the post hoc tests (n = 4–6). AFR: animal facility rearing. Bars: 200 µm.

In the CA1 region, the main effect of maternal care appeared to be significant (F_1,32_ = 11.168; *p* = 0.002). Additionally, a second-order effect of maternal care and stress (F_1,32_ = 9.071; *p* = 0.005) and, more importantly, a maternal care × stress × treatment triple interaction was seen (F_1,32_ = 30.848; *p* < 10^−5^). In AFR mice, exposure to CVMS (bars “a” and “c” in Figure 2E, *p* < 0.001) reduced the H3K9ac immunosignal that was reversed by fluoxetine treatment (bars “c” and “d”, *p* = 0.001). A history of MD180 was associated with lower H3K9ac-ir in the control mice (see bars “a” and “e” in Figure 2E, *p* < 10^−4^). Interestingly, in the CA1 region, fluoxetine treatment (compare bars “e” and “f” in Figure 2E, *p* < 10^−4^, as well as Figure 2A,C) and CVMS exposure (see bars “e” and “g” in Figure 2E, *p* < 0.001, as well as Figure 2A,B) increased the H3K9ac immunosignal. Fluoxetine treatment in the CVMS-exposed MD180 mice did not affect the H3K9ac-ir in the CA1 region (see columns “g” and “h” in Figure 2E, *p* = 0.66, and also images Figure 2B,D).

In the CA3 area, the main effect of maternal care (F_1,32_ = 5.454; *p* = 0.026) and a third-order effect of the maternal care × stress × treatment interaction (F_1,32_ = 29.286; *p* < 10^−5^) influenced the histone acetylation significantly. Again, upon exposure to CVMS (bars “a” and “c” in Figure 2F, *p* = 0.004), AFR mice showed a lower H3K9ac immunosignal, and if they were treated with fluoxetine (bars “c” and “d”, *p* = 0.004), the H3K9ac-ir was higher again. MD180 resulted in a relatively low signal in the vehicle-treated control mice (bars “a” and “e” in Figure 2F, *p* < 10^−4^). Fluoxetine treatment increased the histone acetylation in MD180 control mice (compare bars “e” and “f” in Figure 2F, *p* = 0.001). Exposure to CVMS increased the H3K9ac-ir (see bars “e” and “g”, *p* < 0.001), but if the fluoxetine treatment was administered in MD180 PACAP HZ mice upon CVMS exposure, which was the case that carried all three “hits”, the histone acetylation appeared to be lower (compare bars “g” and “h” in Figure 2F, *p* = 0.008).

In the DG, the main effects of maternal care (F_1,32_ = 30.65; *p* < 10^−5^) and stress (F_1,32_ = 15.25; *p* < 0.001) affected H3K9ac. MANOVA found that the interactions of maternal care × stress (F_1,32_ = 38.14; *p* < 10^−5^), stress × treatment (F_1,32_ = 15.83; *p* < 0.001), as well as maternal care × stress × treatment (F_1,32_ = 74.61; *p* < 10^−6^) influenced the histone acetylation in the DG. In AFR animals, the dynamics of H3K9ac-ir was similar to that we observed in the CA1 and CA3 regions: CVMS exposure reduced the immunosignal (bars “a” and “c” in Figure 2G, *p* < 10^−6^), which was tendentiously reversed by the fluoxetine administration (see bars “c” and “d” Figure 2G, *p* = 0.079). The control, vehicle-treated mice that experienced MD180 showed a reduced level of H3K9ac-ir (compare bars “a” and “e” in Figure 2G, *p* < 10^−6^). The effect of fluoxetine treatment on the H3K9ac-ir depended on CVMS exposure in MD180 mice: in the control animals, fluoxetine treatment elevated the level of H3K9ac-ir (see bars “e” and “f”, *p* < 10^−5^), while in CVMS-exposed mice, the SSRI reduced the histone acetylation (compare bars “g” and “h” in Figure 2G, *p* < 10^−6^).

### 2.2. FOSB Immunoreactivity

The FOSB-ir was also quantified, to determine the neuronal activity in the PFC and the hippocampal areas. The FOSB-ir was successfully detected in the neuronal nuclei both in the PFC and the hippocampus.

#### 2.2.1. Prefrontal Cortex

**Figure 3:**
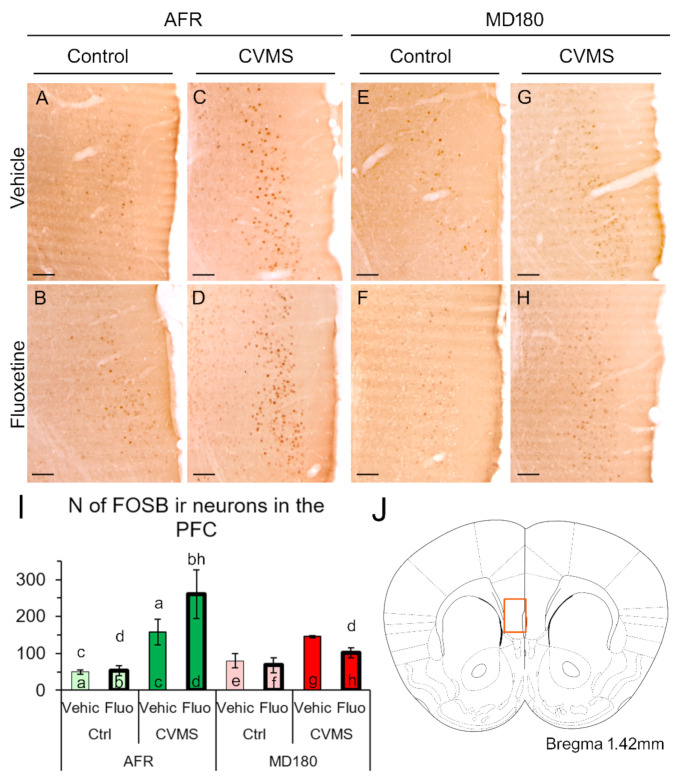
FOSB immunoreactivity (ir) in the prefrontal cortex (PFC). Representative images illustrate FOSB-ir in the PFC of vehicle (Vehic) (**A**) and fluoxetine (Fluo)-treated (**B**) control (Ctrl) as well as chronic variable mild stress-exposed (CVMS) and vehicle- (**C**) or fluoxetine-injected AFR mice. History of maternal deprivation (MD180) affected the FOSB-ir in the respective groups, as illustrated by images (**E**–**H**). The imaged area shown in (**A**–**H**) corresponds to the highlighted region of PFC in (**J**) (scheme modified after Paxinos and Franklin [62]). Histogram (**I**) illustrates the count of FOSB-ir cell nuclei. Lettering indicates the most relevant significant differences between pairs of groups, according to the post hoc tests (n = 4–6). AFR: animal facility rearing. Bars: 100 µm.

A relatively strong neuronal activity was observed in the PFC of all the mouse groups in this experiment. The main effect of stress (F_1,32_ = 28.601; *p* < 10^−4^), and the interaction of maternal care and stress (F_1,32_ = 5.078; *p* = 0.032) exerted a significant effect on FOSB-ir. In AFR mice, CVMS exposure caused a significant elevation of the FOSB-ir in both the vehicle-treated (compare bars “a” and “c” in Figure 3I, *p* < 0.001, and Figure 3A with Figure 3C) and fluoxetine-treated mice (bars “b” and “d” in Figure 3I, *p* < 0.001, as well as panels in Figure 3B,D). If fluoxetine was administered in CVMS-exposed AFR mice (see bar “d” in Figure 3I and image Figure 3D), a higher (*p* < 10^−4^) FOSB cell count was detected, compared with the control fluoxetine-treated AFR mice (see bar “b” in Figure 3I and image Figure 3C). Upon maternal deprivation, neither CVMS nor fluoxetine treatment influenced the FOSB-ir in the PFC (see bars “e-h” in Figure 3I; illustrated also in Figure 3E–H). The count of FOSB-ir cells in the stressed and fluoxetine-treated MD180 mice was much lower (*p* = 0.002) than that observed in the respective AFR groups (see bars “d” and “h” in Figure 3I and images Figure 3D,H).

#### 2.2.2. Hippocampus

The FOSB-ir in the hippocampus was weaker than that observed in the PFC. While in the AFR groups, we detected low cell numbers, in MD180 mice, the signal became almost undetectable in all the subgroups.

**Figure 4:**
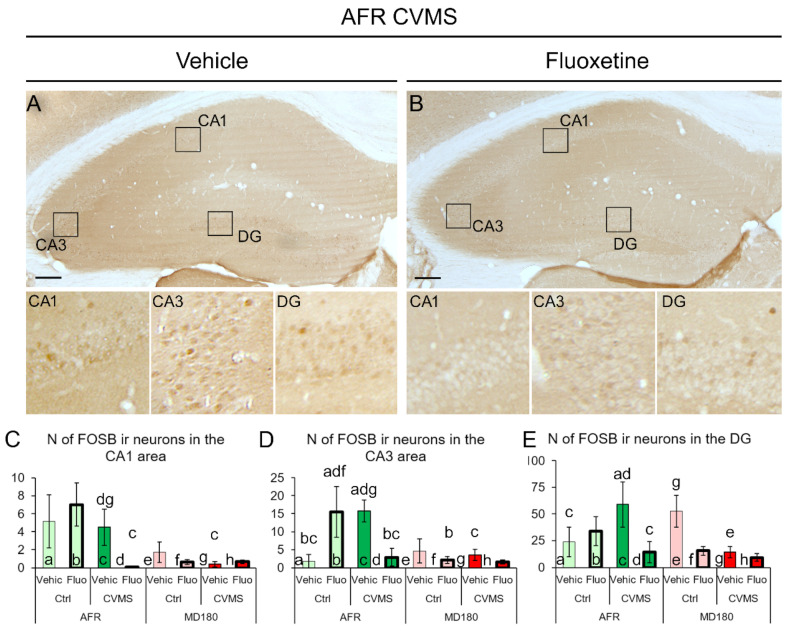
FOSB immunoreactivity (ir) in the cornu ammonis (CA) 1, CA3, and dentate gyrus (DG) of the hippocampus. Representative images illustrating FOSB-ir nuclei in the hippocampus of vehicle (Vehic) (**A**) and fluoxetine (Fluo)-treated (**B**) chronic variable mild stress (CVMS)-exposed animal facility-reared (AFR) mice. Note the relatively weak signal in these groups that became practically undetectable in the hippocampus of mice that earlier experienced maternal deprivation (MD180). Boxed areas are shown in higher magnification insets below the respective low magnification images. Histograms illustrate the count of FOSB-ir cell nuclei in CA1 (**C**), CA3 (**D**), and DG (**E**) of the hippocampus. Lettering indicates the most relevant significant differences between pairs of groups, according to the post hoc tests (n = 4–6). Ctrl: control, Bars: 200 µm.

In the CA1 area, the main effect of maternal care (F_1,32_ = 7.378; *p* = 0.010) and the third-order effect of maternal care × stress × treatment interaction (F_1,32_ = 7.235; *p* = 0.011) affected the FOSB-ir. In CVMS-exposed AFR mice, fluoxetine treatment reduced the FOSB-ir to an almost undetectable level (compare Figure 4A,B moreover bars “c” and “d” in Figure 4C, *p* = 0.009). In vehicle-treated, CVMS-exposed MD180 mice, the FOSB signal was also very low (compare bars “c” and “g”, *p* = 0.027), which could not be further reduced by fluoxetine administration (compare bars “g” and “h” in Figure 4C, *p* = 0.51).

In the CA3 area, ANOVA revealed the main effect of maternal care (F_1,32_ = 5.118; *p* = 0.031), as well as the stress × treatment (F_1,32_ = 11.70; *p* = 0.018) and maternal care × stress × treatment (F_1,32_ = 7.616; *p* = 0.009) interactions, on FOSB-ir to be significant. In the control AFR mice, fluoxetine increased the FOSB cell count (compare bars “a” and “b” in Figure 4D, *p* = 0.012). CVMS exposure also increased the FOSB signal in AFR mice (compare bars “a” and “c” in Figure 4D, *p* = 0.008), while fluoxetine reduced this to the basal level (see bars “c” and “d” in Figure 4D, *p* = 0.008 and images Figure 4A,B). A history of MD180 exposure resulted in the complete absence of FOSB response to fluoxetine (compare bars “b” and “f” in Figure 4D, *p* = 0.010) or CVMS exposure (see columns “c” and “g” in Figure 4D, *p* = 0.034). 

In the DG, the main effect of treatment (F_1,32_ = 7.086; *p* = 0.013) and the triple interaction of maternal care, stress, and treatment (F_1,32_ = 9.267; *p* = 0.005) influenced the FOSB signal significantly. In AFR mice, stress exposure increased the FOSB cell count (compare bars “a” and “c” in Figure 4E, *p* = 0.042), which was reversed by fluoxetine treatment (see columns “c” and “d” in Figure 4E, *p* = 0.002 and also images Figure 4A,B). In MD180 mice, there was some FOSB immunosignal detectable in vehicle-treated control mice (bar “e” in Figure 4E), but if MD180 mice were exposed to CVMS, the FOSB remained undetectable (compare bars “e” and “g” in Figure 4E, *p* = 0.032), which could not be further decreased by fluoxetine administration (see bars “g” and “h”, *p* = 0.43).

## 3. Discussion

In our recent studies, we demonstrated that our three-hit-theory-based animal model for depression fulfills the Willnerian construct and the face and predictive validity criteria including (a) the behavioral anomalies as assessed in depression and anxiety tests, (b) the functional–neuromorphological alterations in multiple limbic areas, and (c) the altered physical and endocrinological measures of the stress effect [6,21]. Here, we aimed to examine the functional–morphological changes in the PFC and hippocampus focusing on the epigenetic marker H3K9ac, and on the chronic neuronal activity indicator FOSB. Our results suggested that both limbic regions might contribute to the behavioral changes [5,6,21] observed in this model, as discussed below.

### 3.1. The H3K9ac-ir Is Affected by Both Maternal Deprivation and Fluoxetine Therapy

Histone acetylation is one of the commonly examined types of epigenetic modifications that do not affect the DNA sequence proper, but contribute to the control of gene expression [63]. The level of acetylation is determined by the dynamics of histone acetyltransferase and histone deacetylase enzymes [64]. In the PFC, we found that in AFR mice, fluoxetine treatment was associated with reduced H3K9ac-ir, but if the mice underwent MD180, a lower H3K9ac level was seen that was not affected by fluoxetine therapy. This suggests that fluoxetine therapy and early life stress interact in the PFC, which is in line with an earlier study [65]. However, in contrast to our findings, Levine et al. [65] found that fluoxetine therapy potentiated epigenetic changes. This discrepancy may be explained by the difference in the examined histone acetylation site at histone H4. Meanwhile, in another laboratory, Robinson et al. [66] also found that chronic fluoxetine exposure reduced epigenetic changes in the nucleus accumbens.

The dynamics of the hippocampal H3K9ac-ir differed from that in the PFC. MD180 reduced histone H3 acetylation in all the hippocampal divisions, which is in agreement with the work of Sun et al. [58]. CVMS reduced the H3K9ac-ir in the vehicle-treated AFR mice in all the examined hippocampal subdivisions. This is in agreement with an earlier rat study [59], except for the CA1, where no change was found by Ferland and Schrader [59]. Fluoxetine increased the H3K9ac-ir in mice that earlier experienced CVMS. This is well-comparable with the findings by Hunter et al. [60], according to which the methylation of this histone was evaluated. Our present study showed that in the CVMS-exposed mice that also suffered MD180, the acetylation-increasing effect of fluoxetine was abolished in the CA1 and was inverted into a decreasing effect in the CA3 and DG. This, in our model, suggests that the efficacy of antidepressants may depend on the number of risk factors and the epigenetic status that the model animal model carries. This ultimately may underline the importance of the individualized therapeutic approach in the management of depression to increase the efficacy of pharmacotherapy.

### 3.2. FOSB Reactivity to CVMS Is Influenced Both by Maternal Care Quality and SSRI Treatment

FOSB is a commonly used neuronal activity marker in stress research reflecting that the cellular response to a particular stimulus requires a response at the level of gene transcription [6,9,47,67,68,69,70]. The antibody used here recognizes both the full-length FOSB and its variant, the deltaFOSB [70], both of which contribute to the transcription factor activator protein 1 complex. These two isoforms differ in their dynamics: Full-length FOSB displays a faster acute response to stress exposure, but it is eliminated in a shorter period of time [47]. In contrast, if the stimulus was repeated, the deltaFOSB accumulates in the cells and can be detected even after a week [47]. Considering the shorter half-life of the full-length FOSB, and because in this study, the mice were perfused one day after the last stress exposure, the detected FOSB protein signal should correspond to the delta isoform, which mirrors chronic neuronal activation [47,62,65,69,71].

The phenomenon that chronic stress exposure increases the FOSB-ir in the PFC is well-known [62,71,72,73], and it is in full agreement with our findings in the vehicle-treated CVMS-exposed mice. Fluoxetine treatment per se was also shown to increase the FOSB content in the PFC [74]; however, in the present work, this increase remained below the significant level, which could be explained by the shorter treatment period (i.e., 14 vs. 20 days). Importantly, we observed here that if CVMS exposure was superimposed on the history of MD180, neither CVMS nor fluoxetine therapy elevated the FOSB activity in the PFC. This phenomenon may have great translational relevance, as FOSB-related transcriptional changes were suggested to determine therapeutic efficacy in the management of major depression [74].

As the history of MD180 was associated with a very low, almost undetectable FOSB signal in the hippocampus, it has to be stated that there was not much space for the SSRI treatment to reduce the FOSB-ir. One may argue that a technical error may have occurred that prevented the immunolabeling, but because the PFC sections of the same animals in the same staining process gave a reliable signal, we assume that the low hippocampal FOSB-ir in MD180 mice is a true area-specific observation. An alternative interpretation of this phenomenon could be that the effect of the fluoxetine treatment cannot be detected by FOSB labeling upon MD180, but other markers may still mirror the effect of SSRI treatment [41,42,43]. Nevertheless, this idea is also supported by our findings in the H3K9ac labeling.

### 3.3. Limitations

Taking the complexity of the model and the capacity limitations of our animal facility into consideration, we had to restrict the study to male animals. Considering that the examined brain areas are estrogen-sensitive [75,76], the effect of the estrous cycle could have influenced the results by increasing the error due to the random estrous cycle phase of the mice at the time of tissue collection. A regular follow-up examination of the cycle phase in mice by collecting vaginal smears would have also caused an additional stress factor. Therefore, it is a true limitation of this study that we do not know whether the present findings would be characteristic of the examined brain regions in female animals.

In this study, we did not examine the histochemical characteristics of the cells that gave a positive signal for H3K9ac and FOSB. Further co-localization studies have to determine if there is a cell-type-specific alteration in the histone acetylation or neuronal activity pattern in the PFC and the hippocampal subdivisions. Considering that for some types of neurons, the expression of the neuronal activity marker immediate early genes is not characteristic [77,78], we cannot rule out that the pattern of the neuronal activity would show a different picture if we had used an alternative marker instead of FOSB. The other disadvantage of FOSB labeling is that it does not detect potentially very important functional changes with inhibitory nature [79,80], suggesting that the reduced activity and/or gene expression in the examined areas may have occurred. Based on the current findings, further investigations are required to examine how neuroinflammatory mechanisms contribute to the functional changes in the PFC and the hippocampus. Additionally, multiple other indicators of stress effect may be examined in the PFC and the hippocampus, including neurotrophins [81,82] and neurogenesis [83], to further dissect the underlying mechanisms.

Several lines of epigenetic markers have been identified in the past [43,84], and this study was restricted to H3K9ac. In order to gain deeper insight into possible changes at the gene expression level, the examination of multiple markers would be beneficial, and in some selected cell types, promoter-specific studies would be required to test how they are affected in this model [70].

### 3.4. Conclusions

With respect to the limitations of this study, our three-hit concept-based mouse model for depression in male PACAP HZ mice [5,6,21] reproducibly fulfills the Willnerian criteria [28] of a reliable animal model in male mice at the behavioral, endocrinological [21] and functional–morphological level. In this study, we showed that the pattern of both H3K9ac and FOSB-ir is modulated in a brain-area-specific manner by early life stress and chronic stress in later life, and they interact with the effect of fluoxetine therapy. Considering the complex epigenetic and neuronal activity changes in our model, and taking the significance of the PFC and the hippocampus [31] in mood control into consideration, we conclude that these limbic centers might contribute to the depression-like phenotype [21], further supporting the reliability of our model in male mice.

## 4. Materials and Methods

### 4.1. Animals and Experimental Design

The experimental design corresponds to our most recently published works [6,21]. Briefly, male and female PACAP HZ mice were paired, and seventeen litters born within 3 days were used. Litter differences were prevented by cross-fostering on postnatal day (PD) 1. Seven dams with the offspring were subjected to normal animal facility rearing (AFR) protocol. In the case of 10 litters, on PD 1-14, the dam was removed for 180 minutes in order to cause maternal deprivation (MD180). During this period, the pups with the nesting material were placed on a heating plate at 32 °C. The adult offspring was tail-clipped on PD 70, for genotyping via PCR (for details, see [8]).

In total, 17 PACAP HZ male mice were identified in the AFR litters, while 25 PACAP HZ male mice were found in the MD180 litters. We did not use the female offspring in this study, and we also excluded the wild-type and PACAP KO mice, based on our earlier findings [5,6,21].

Both AFR and MD180 mice were randomly assigned to four subgroups, as shown in Table 1. We exposed four subgroups (c, d, and g, h, in Table 1) of the adult offspring to CVMS between PD125 and PD139. In the CVMS protocol, we applied both a mid-day stressor (i.e., tilted cage placement, exposure to a dark room, or placing the cage of the animals on an orbital laboratory shaker) and a challenge in the dark phase (wet nesting material, individual caging of mice as social isolation, or group holding). As controls to the CVMS, four subgroups (a, b, and e, f in Table 1) were left undisturbed. For further technical details on the CVMS protocol, see [5,6,21].

Half of the subgroups (b, d, f, h) were treated with intraperitoneal (ip) fluoxetine (20 mg/kg/day in 0.2 mL saline during the 14 days period of CVMS exposure) injections. The mice in subgroups a, c, e, and g received physiological saline (0.2 mL vehicle ip) injections. Each group (i.e., a–h) consisted of 4–6 animals.

The mice were kept under standard housing conditions (24 °C, 50% air humidity, 12/12-hour light–dark phases with lights on from 6 am), at the animal facility of the Department of Anatomy, the University of Pécs, in standard (30 cm × 30 cm × 28 cm) polycarbonate cages with 4–5 mice per cage group. The mice had ad libitum access to drinking water and normal standard rodent chow and were placed on fresh nesting material in a clean cage every other day.

### 4.2. Perfusion and Sample Preparation

In this work, the stored brain tissue samples collected in our most recent study [21] were used. The mice were quickly euthanized using an overdose of urethane injection (ip; 2.4 mg/kg) on PD140, and they were transcardially perfused with 20 mL of 0.1 M phosphate-buffered saline (PBS, pH 7.4) and with 150 mL 4% paraformaldehyde in a Millonig buffer (pH 7.4) for 20 min [85]. Subsequently, the brains were collected, postfixed, and coronally sectioned using a vibratome (Leica VT1000 S, Leica Biosystems, Wetzlar, Germany). Then, 30 µm thick sections were collected in four representative series. Free-floating sections were immersed and stored in an anti-freeze solution at −20 °C until labeling.

Immunolabeling was performed on manually selected sections based on the *Mouse Brain Atla*s by Paxinos and Franklin [62]. The brain sections between Bregma +1.34 mm and +1.94 mm were used to study the PFC. The coronal planes between Bregma −1.58 mm and −2.18 mm were selected to examine the hippocampus.

### 4.3. Free-Floating Immunocytochemistry for H3K9ac and FOSB by Diaminobenzidine

The sections were rinsed 4 × 15 min in PBS, permeabilized with 0.5% Triton X-100 for 1 hour, and blocked with 2% normal goat serum (NGS) (Jackson Immunoresearch). Then, the sections were placed into a solution of 2% NGS containing anti-acetyl-lysine 9 H3 histone antibodies (1:4000, Sigma-Aldrich; Cat# SAB4500347; RRID: AB_10742909) or into a 2% NGS solution of rabbit anti-FOSB antiserum (1:500, Santa Cruz, sc-48, RRID: AB_631515, Santa Cruz Biotechnology Inc., Santa Cruz CA, USA), followed by overnight incubation at 20 °C. Upon washes in PBS, a biotinylated goat anti-rabbit IgG solution was applied for 1 hour (diluted to 1:200, Vectastain ABC Elite Kit, Vector Lbs., Burlingame, CA, USA). Subsequently, after rinsing in PBS, the preparations were treated for 1 hour with a peroxidase-conjugated avidin–biotin complex (Vectastain ABC Elite Kit). After further PBS washes, the antibody binding was visualized in a Tris buffer (pH 7.4) containing 0.02% 3,3′diaminobenzidine (DAB, Sigma-Aldrich) and 0.03% (*w*/*v*) H_2_O_2_. The reaction was controlled by visual observation in a light microscope and stopped in PBS after 7 min. After several PBS washes, the preparations were mounted on gelatinized slides, air-dried, and dehydrated in ethanol solutions (50%, 70%, 96%, absolute ethanol, 5 min, respectively). After clearing in xylene (2 × 10 min), the sections were cover-slipped with Depex (Fluka, Heidelberg, Germany).

The H3K9ac antiserum was raised in the rabbit using a synthetic peptide (range of residues 3-52), including the acetylation site at Lys9 in histone H3. The manufacturer published that the serum is specific to mice (https://www.sigmaaldrich.com/catalog/product/sigma/sab4500347?lang=hu&region=HU, accessed on 1 October 2022). The FOSB serum (Santa Cruz, sc-48) was generated also in the rabbit, immunized with a C-terminal part of human FOSB protein. This serum was also characterized earlier by our group [6,9,21,70]. The omission of the primary and secondary serum and their replacement with normal serum did not give any recognizable immunosignal in the randomly selected sections of the tissue samples examined in this study. The preabsorption of the antibodies to the synthetic blocking peptides abolished the immunosignal [6,9,21].

### 4.4. Microscopy, Digital Imaging, and Morphometry

Microscopic preparations were evaluated and digitalized with a Nikon Microphot FXA microscope using a Spot RT camera (Nikon, Tokyo, Japan). From all mouse brains, five selected coronal sections of the PFC and the hippocampus were imaged. The count of immunoreactive nuclei was determined using the manual cell counting multipoint tool of ImageJ software (v1.42, NIH, Bethesda, MD) in non-edited images. All data were collected by an expert neurohistologist colleague who was not informed about the identity of the preparations.

In the case of the PFC, the whole cross-sectional surface area of the PFC in an area of 500 µm × 750 µm (Figure 1C) was evaluated. In the case of the hippocampus, the immunoreactive nuclei were counted in a 1200 µm range in the pyramidal layer of CA1 and CA3 regions. In the DG, a 1200 µm range of the granular layer was evaluated. The cell counts were averaged from five digital images per brain area, and this number represented the brain area of one animal in the statistics.

For publication purposes, the selected representative images were contrasted, cropped, and edited into photo montages using Photoshop software (Adobe, San Jose, CA, USA).

### 4.5. Statistics

All data are presented as the mean of the group. The error bars depict the standard error of the mean in all graphs. The statistical evaluation was carried out using Statistica software (v8.0; StatSoft, Tulsa, OK, USA). Few data points beyond the two sigma range were excluded from the assessment. After testing for normality by Shapiro–Wilk test [86] and homogeneity using Bartlett’s chi-square test [87], the data were subjected to a multifactorial analysis of variance (MANOVA) with the categorical predictors “maternal care”, “stress”, and “treatment”. In the case of significant main effects and/or interactions, the differences between the pairs of groups were further assessed by Fisher’s post hoc tests (α < 5%). A logarithmic data transformation was applied if the datasets did not show normal distribution.

## Figures and Tables

**Table 1:** Experimental design. Lettering (a–h) and the background color of cells in the “group” row correspond to the labeling of bars in Figure 1, Figure 2, Figure 3 and Figure 4. Green: animal-facility-reared (AFR) groups; red: groups with history of maternal deprivation (MD180). Control (light shade) and chronic variable mild stress (CVMS)-exposed (dark shade) groups were subdivided into vehicle- and fluoxetine-treated subgroups (thick black frames).

	AFR				MD180			
	Control		CVMS		Control		CVMS	
	Vehicle	Fluoxetine	Vehicle	Fluoxetine	Vehicle	Fluoxetine	Vehicle	Fluoxetine
group	a	b	c	d	e	f	g	h

## Data Availability

The raw data collected in this study are available upon request from the corresponding author.

## References

[1] (2021). WHO. https://www.who.int/news-room/fact-sheets/detail/depression.

[2] Park C., Rosenblat J.D., Brietzke E., Pan Z., Lee Y., Cao B., Zuckerman H., Kalantarova A., McIntyre R.S. (2019). Stress, epigenetics and depression: A systematic review. Neurosci. Biobehav. Rev..

[3] De Kloet E.R., DeRijk R.H., Meijer O.C. (2007). Therapy insight: Is there an imbalanced response of mineralocorticoid and glucocorticoid receptors in depression?. Nat. Clin. Pract. Endocrinol. Metab..

[4] Daskalakis N.P., Bagot R.C., Parker K.J., Vinkers C.H., de Kloet E.R. (2013). The three-hit concept of vulnerability and resilience: Toward understanding adaptation to early-life adversity outcome. Psychoneuroendocrinology.

[5] Farkas J., Kovacs L.Á., Gaszner T., Gaszner B., Reglődi D., Tamás A. (2016). Using PACAP heterozygous mice as models of the three hit theory of depression. Pituitary Adenylate Cyclase Activating Polypeptide—PACAP.

[6] Farkas J., Kovács L., Gáspár L., Nafz A., Gaszner T., Ujvári B., Kormos V., Csernus V., Hashimoto H., Reglődi D. (2017). Construct and face validity of a new model for the three-hit theory of depression using PACAP mutant mice on CD1 background. Neuroscience.

[7] Hashimoto H., Shintani N., Tanida M., Hayata A., Hashimoto R., Baba A. (2011). PACAP is Implicated in the Stress Axes. Curr. Pharm. Des..

[8] Gaszner B., Kormos V., Kozicz T., Hashimoto H., Reglodi D., Helyes Z. (2012). The behavioral phenotype of pituitary adenylate-cyclase activating polypeptide-deficient mice in anxiety and depression tests is accompanied by blunted c-Fos expression in the bed nucleus of the stria terminalis, central projecting Edinger–Westphal nucleus, ventral lateral septum, and dorsal raphe nucleus. Neuroscience.

[9] Kormos V., Gáspár L., Kovács L., Farkas J., Gaszner T., Csernus V., Balogh A., Hashimoto H., Reglődi D., Helyes Z. (2016). Reduced response to chronic mild stress in PACAP mutant mice is associated with blunted FosB expression in limbic forebrain and brainstem centers. Neuroscience.

[10] Agarwal A., Halvorson L.M., Legradi G. (2005). Pituitary adenylate cyclase-activating polypeptide (PACAP) mimics neuroendocrine and behavioral manifestations of stress: Evidence for PKA-mediated expression of the corticotropin-releasing hormone (CRH) gene. Mol. Brain Res..

[11] Stroth N., Eiden L. (2010). Stress hormone synthesis in mouse hypothalamus and adrenal gland triggered by restraint is dependent on pituitary adenylate cyclase-activating polypeptide signaling. Neuroscience.

[12] Stroth N., Holighaus Y., Ait-Ali D., Eiden L.E. (2011). PACAP: A master regulator of neuroendocrine stress circuits and the cellular stress response. Ann. N. Y. Acad. Sci..

[13] Tsukiyama N., Saida Y., Kakuda M., Shintani N., Hayata A., Morita Y., Tanida M., Tajiri M., Hazama K., Ogata K. (2011). PACAP centrally mediates emotional stress-induced corticosterone responses in mice. Stress.

[14] Pinhasov A., Nesher E., Gross M., Turgeman G., Kreinin A., Yadid G. (2011). The Role of the PACAP Signaling System in Depression. Curr. Pharm. Des..

[15] Reglodi D., Kiss P., Lubics A., Tamas A. (2011). Review on the Protective Effects of PACAP in Models of Neurodegenerative Diseases In Vitro and In Vivo. Curr. Pharm. Des..

[16] Reglodi D., Kiss P., Szabadfi K., Atlasz T., Gabriel R., Horvath G., Szakaly P., Sandor B., Lubics A., Laszlo E. (2012). PACAP is an Endogenous Protective Factor—Insights from PACAP-Deficient Mice. J. Mol. Neurosci..

[17] Kormos V., Gaszner B. (2013). Role of neuropeptides in anxiety, stress, and depression: From animals to humans. Neuropeptides.

[18] Hammack S.E., May V. (2015). Pituitary Adenylate Cyclase Activating Polypeptide in Stress-Related Disorders: Data Convergence from Animal and Human Studies. Biol. Psychiatry.

[19] Lutfy K., Shankar G. (2019). Emerging evidence for the role of pituitary adenylate cyclase-activating peptide in neuropsychiatric disorders. Prog. Mol. Biol. Transl. Sci..

[20] Boucher M.N., May V., Braas K.M., Hammack S.E. (2021). PACAP orchestration of stress-related responses in neural circuits. Peptides.

[21] Gaszner T., Farkas J., Kun D., Ujvári B., Berta G., Csernus V., Füredi N., Kovács L., Hashimoto H., Reglődi D. (2022). Fluoxetine treatment supports predictive validity of the three hit model of depression in male PACAP heterozygous mice and underpins the impact of early life adversity on therapeutic efficacy. Front. Endocrinol..

[22] Lange U.C., Schneider R. (2010). What an epigenome remembers. BioEssays.

[23] Ng R.K., Gurdon J.B. (2008). Epigenetic inheritance of cell differentiation status. Cell Cycle.

[24] Nestler E.J. (2012). Stress makes its molecular mark. Nature.

[25] Raabe F.J., Spengler D. (2013). Epigenetic Risk Factors in PTSD and Depression. Front. Psychiatry.

[26] Heim C., Binder E.B. (2012). Current research trends in early life stress and depression: Review of human studies on sensitive periods, gene–environment interactions, and epigenetics. Exp. Neurol..

[27] Willner P. (2017). The chronic mild stress (CMS) model of depression: History, evaluation and usage. Neurobiol. Stress.

[28] Willner P. (1984). The validity of animal models of depression. Psychopharmacology.

[29] Liu W., Ge T., Leng Y., Pan Z., Fan J., Yang W., Cui R. (2017). The Role of Neural Plasticity in Depression: From Hippocampus to Prefrontal Cortex. Neural Plast..

[30] Belleau E.L., Treadway M.T., Pizzagalli D.A. (2019). The Impact of Stress and Major Depressive Disorder on Hippocampal and Medial Prefrontal Cortex Morphology. Biol. Psychiatry.

[31] Treadway M.T., Waskom M.L., Dillon D.G., Holmes A., Park M.T., Chakravarty M.M., Dutra S.J., Polli F.E., Iosifescu D.V., Fava M. (2015). Illness Progression, Recent Stress, and Morphometry of Hippocampal Subfields and Medial Prefrontal Cortex in Major Depression. Biol. Psychiatry.

[32] George M.S., Ketter T.A., Post R.M. (1994). Prefrontal cortex dysfunction in clinical depression. Depression.

[33] Pizzagalli D.A., Roberts A.C. (2022). Prefrontal cortex and depression. Neuropsychopharmacology.

[34] Mayberg H.S., Liotti M., Brannan S.K., McGinnis S., Mahurin R.K., Jerabek P.A., Silva J.A., Tekell J.L., Martin C.C., Lancaster J.L. (1999). Reciprocal limbic-cortical function and negative mood: Converging PET findings in depression and normal sadness. Am. J. Psychiatry.

[35] Ellenbogen J.M., Hurford M.O., Liebeskind D.S., Neimark G.B., Weiss D. (2005). Ventromedial frontal lobe trauma. Neurology.

[36] Mayberg H.S., Lozano A.M., Voon V., McNeely H.E., Seminowicz D., Hamani C., Schwalb J.M., Kennedy S.H. (2005). Deep brain stimulation for treatment-resistant depression. Neuron.

[37] Koenigs M., Grafman J. (2009). The functional neuroanatomy of depression: Distinct roles for ventromedial and dorsolateral prefrontal cortex. Behav. Brain Res..

[38] Albert P.R., Vahid-Ansari F., Luckhart C. (2014). Serotonin-prefrontal cortical circuitry in anxiety and depression phenotypes: Pivotal role of pre- and post-synaptic 5-HT1A receptor expression. Front. Behav. Neurosci..

[39] Ghosal S., Duman C.H., Liu R.-J., Wu M., Terwilliger R., Girgenti M.J., Wohleb E., Fogaca M.V., Teichman E.M., Hare B. (2020). Ketamine rapidly reverses stress-induced impairments in GABAergic transmission in the prefrontal cortex in male rodents. Neurobiol. Dis..

[40] Veeraiah P., Noronha J.M., Maitra S., Bagga P., Khandelwal N., Chakravarty S., Kumar A., Patel A.B. (2014). Dysfunctional Glutamatergic and γ-Aminobutyric Acidergic Activities in Prefrontal Cortex of Mice in Social Defeat Model of Depression. Biol. Psychiatry.

[41] Blendy J.A. (2006). The role of CREB in depression and antidepressant treatment. Biol. Psychiatry.

[42] Yan L., Xu X., He Z., Wang S., Zhao L., Qiu J., Wang D., Gong Z., Qiu X., Huang H. (2020). Antidepressant-Like Effects and Cognitive Enhancement of Coadministration of Chaihu Shugan San and Fluoxetine: Dependent on the BDNF-ERK-CREB Signaling Pathway in the Hippocampus and Frontal Cortex. BioMed Res. Int..

[43] Dionisie V., Ciobanu A.M., Toma V.A., Manea M.C., Baldea I., Olteanu D., Sevastre-Berghian A., Clichici S., Manea M., Riga S. (2021). Escitalopram Targets Oxidative Stress, Caspase-3, BDNF and MeCP2 in the Hippocampus and Frontal Cortex of a Rat Model of Depression Induced by Chronic Unpredictable Mild Stress. Int. J. Mol. Sci..

[44] Baudin A., Blot K., Verney C., Estevez L., Santamaria J., Gressens P., Giros B., Otani S., Daugé V., Naudon L. (2012). Maternal deprivation induces deficits in temporal memory and cognitive flexibility and exaggerates synaptic plasticity in the rat medial prefrontal cortex. Neurobiol. Learn. Mem..

[45] Hinwood M., Morandini J., Day T.A., Walker F.R. (2012). Evidence that Microglia Mediate the Neurobiological Effects of Chronic Psychological Stress on the Medial Prefrontal Cortex. Cereb. Cortex.

[46] Kovács K.J. (2008). Measurement of immediate-early gene activation-c-fos and beyond. J. Neuroendocrinol..

[47] Nestler E.J. (2015). ∆FosB: A transcriptional regulator of stress and antidepressant responses. Eur. J. Pharmacol..

[48] Sampath D., Sathyanesan M., Newton S.S. (2017). Cognitive dysfunction in major depression and Alzheimer’s disease is associated with hippocampal–prefrontal cortex dysconnectivity. Neuropsychiatr. Dis. Treat..

[49] Campbell S., MacQueen G. (2004). The role of the hippocampus in the pathophysiology of major depression. J. Psychiatry Neurosci..

[50] Xu L., Anwyl R., Rowan M.J. (1997). Behavioural stress facilitates the induction of long-term depression in the hippocampus. Nature.

[51] Pittenger C., Duman R.S. (2008). Stress, Depression, and Neuroplasticity: A Convergence of Mechanisms. Neuropsychopharmacology.

[52] Czéh B., Michaelis T., Watanabe T., Frahm J., de Biurrun G., van Kampen M., Bartolomucci A., Fuchs E. (2001). Stress-induced changes in cerebral metabolites, hippocampal volume, and cell proliferation are prevented by antidepressant treatment with tianeptine. Proc. Natl. Acad. Sci. USA.

[53] Czéh B., Lucassen P.J. (2007). What causes the hippocampal volume decrease in depression?. Eur. Arch. Psychiatry Clin. Neurosci..

[54] Masi G., Brovedani P. (2011). The Hippocampus, Neurotrophic Factors and Depression. CNS Drugs.

[55] Fuchs E., Czéh B., Kole M., Michaelis T., Lucassen P.J. (2004). Alterations of neuroplasticity in depression: The hippocampus and beyond. Eur. Neuropsychopharmacol..

[56] MacQueen G.M., Ramakrishnan K., Ratnasingan R., Chen B., Young L.T. (2003). Desipramine treatment reduces the long-term behavioural and neurochemical sequelae of early-life maternal separation. Int. J. Neuropsychopharmacol..

[57] Zhong H., Rong J., Zhu C., Liang M., Li Y., Zhou R. (2020). Epigenetic Modifications of GABAergic Interneurons Contribute to Deficits in Adult Hippocampus Neurogenesis and Depression-Like Behavior in Prenatally Stressed Mice. Int. J. Neuropsychopharmacol..

[58] Sun H., Zhang X., Kong Y., Gou L., Lian B., Wang Y., Jiang L., Li Q., Sun H., Sun L. (2021). Maternal Separation-Induced Histone Acetylation Correlates with BDNF-Programmed Synaptic Changes in an Animal Model of PTSD with Sex Differences. Mol. Neurobiol..

[59] Ferland C., Schrader L. (2011). Regulation of histone acetylation in the hippocampus of chronically stressed rats: A potential role of sirtuins. Neuroscience.

[60] Hunter R.G., McCarthy K.J., Milne T.A., Pfaff D.W., McEwen B.S. (2009). Regulation of hippocampal H3 histone methylation by acute and chronic stress. Proc. Natl. Acad. Sci. USA.

[61] Perrotti L.I., Hadeishi Y., Ulery P.G., Barrot M., Monteggia L., Duman R.S., Nestler E.J. (2004). Induction of ΔFosB in reward-related brain structures after chronic stress. J. Neurosci..

[62] Paxinos G., Franklin K.B.J. (2001). The Mouse Brain in Stereotaxic Coordinates.

[63] Kouzarides T. (2007). Chromatin modifications and their function. Cell.

[64] Kuo M.H., Allis C.D. (1998). Roles of histone acetyltransferases and deacetylases in gene regulation. Bioessays.

[65] Levine A., Worrell T.R., Zimnisky R., Schmauss C. (2012). Early life stress triggers sustained changes in histone deacetylase expression and histone H4 modifications that alter responsiveness to adolescent antidepressant treatment. Neurobiol. Dis..

[66] Robison A., Vialou V., Sun H.-S., LaBonte B., Golden S., Dias C., Turecki G., Tamminga C.A., Russo S., Mazei-Robison M. (2014). Fluoxetine Epigenetically Alters the CaMKIIα Promoter in Nucleus Accumbens to Regulate ΔFosB Binding and Antidepressant Effects. Neuropsychopharmacology.

[67] Füredi N., Nagy Á., Mikó A., Berta G., Kozicz T., Pétervári E., Balaskó M., Gaszner B. (2017). Melanocortin 4 receptor ligands modulate energy homeostasis through urocortin 1 neurons of the centrally projecting Edinger-Westphal nucleus. Neuropharmacology.

[68] Kovács L., Berta G., Csernus V., Ujvári B., Füredi N., Gaszner B. (2019). Corticotropin-Releasing Factor-Producing Cells in the Paraventricular Nucleus of the Hypothalamus and Extended Amygdala Show Age-Dependent FOS and FOSB/DeltaFOSB Immunoreactivity in Acute and Chronic Stress Models in the Rat. Front. Aging Neurosci..

[69] Kovács L.Á., Füredi N., Ujvári B., Golgol A., Gaszner B. (2022). Age-Dependent FOSB/ΔFOSB Response to Acute and Chronic Stress in the Extended Amygdala, Hypothalamic Paraventricular, Habenular, Centrally-Projecting Edinger-Westphal, and Dorsal Raphe Nuclei in Male Rats. Front. Aging Neurosci..

[70] Sterrenburg L., Gaszner B., Boerrigter J., Santbergen L., Bramini M., Elliott E., Chen A., Peeters B.W.M.M., Roubos E.W., Kozicz T. (2011). Chronic Stress Induces Sex-Specific Alterations in Methylation and Expression of Corticotropin-Releasing Factor Gene in the Rat. PLoS ONE.

[71] Perrotti L., Weaver R., Robison B., Renthal W., Maze I., Yazdani S., Elmore R., Knapp D., Selley D., Martin B. (2008). Distinct patterns of ΔFosB induction in brain by drugs of abuse. Synapse.

[72] Lehmann M.L., Herkenham M. (2011). Environmental Enrichment Confers Stress Resiliency to Social Defeat through an Infralimbic Cortex-Dependent Neuroanatomical Pathway. J. Neurosci..

[73] Laine M., Sokolowska E., Dudek M., Callan S.-A., Hyytiä P., Hovatta I. (2017). Brain activation induced by chronic psychosocial stress in mice. Sci. Rep..

[74] Vialou V., Thibault M., Kaska S., Cooper S., Gajewski P., Eagle A., Mazei-Robison M., Nestler E.J., Robison A. (2015). Differential induction of FosB isoforms throughout the brain by fluoxetine and chronic stress. Neuropharmacology.

[75] Shansky R.M., Hamo C., Hof P.R., Lou W., McEwen B.S., Morrison J.H. (2010). Estrogen Promotes Stress Sensitivity in a Prefrontal Cortex-Amygdala Pathway. Cereb. Cortex.

[76] Spencer J.L., Waters E.M., Romeo R.D., Wood G.E., Milner T.A., McEwen B.S. (2008). Uncovering the mechanisms of estrogen effects on hippocampal function. Front. Neuroendocr..

[77] Dragunow M., Faull R. (1989). The use of c-fos as a metabolic marker in neuronal pathway tracing. J. Neurosci. Methods.

[78] Hoffman G.E., Le W.-W., Abbud R., Lee W.-S., Smith M.S. (1994). Use of Fos-related antigens (FRAs) as markers of neuronal activity: FRA changes in dopamine neurons during proestrus, pregnancy and lactation. Brain Res..

[79] Bowers G., Cullinan W.E., Herman J. (1998). Region-Specific Regulation of Glutamic Acid Decarboxylase (GAD) mRNA Expression in Central Stress Circuits. J. Neurosci..

[80] Choi D.C., Furay A.R., Evanson N.K., Ostrander M.M., Ulrich-Lai Y.M., Herman J.P. (2007). Bed Nucleus of the Stria Terminalis Subregions Differentially Regulate Hypothalamic-Pituitary-Adrenal Axis Activity: Implications for the Integration of Limbic Inputs. J. Neurosci..

[81] Troubat R., Barone P., Leman S., Desmidt T., Cressant A., Atanasova B., Brizard B., El Hage W., Surget A., Belzung C. (2021). Neuroinflammation and depression: A review. Eur. J. Neurosci..

[82] Dwivedi Y. (2009). Brain-derived neurotrophic factor: Role in depression and suicide. Neuropsychiatr. Dis. Treat..

[83] Snyder J.S., Soumier A., Brewer M., Pickel J., Cameron H.A. (2011). Adult hippocampal neurogenesis buffers stress responses and depressive behaviour. Nature.

[84] Saavedra K., Molina-Márquez A.M., Saavedra N., Zambrano T., Salazar L.A. (2016). Epigenetic Modifications of Major Depressive Disorder. Int. J. Mol. Sci..

[85] Szabó T., Kormos V., Rékási Z., Gaszner B. (2021). Epineural Methylene Blue Injection May Aid Localization of Digital Nerves in Dupuytren’s Surgery. Eur. Surg. Res..

[86] Shapiro S.S., Wilk M.B. (1965). An analysis of variance test for normality (complete samples). Biometrika.

[87] Snedecor G.W., Cochran W.G. (1989). Statistical Methods.

